# Supplementary material for: Identification of JPX-RABEP1 Pair as an Immune-Related Biomarker and Therapeutic Target in Pulmonary Arterial Hypertension by Bioinformatics and Experimental Analyses
Source: Int J Mol Sci. 2022 Dec 8;23(24):15559. doi: 10.3390/ijms232415559 (PMC9779127; doi:10.3390/ijms232415559)
Supplement: Supplementary file 1 [file ijms-23-15559-s001.zip › Supplement S5 Primer sequences.pdf]

Supplement S5 Primer sequences

Primer	Sequences
Gapdh-m-F	AGGTCGGTGTGAACGGATTTG
Gapdh-m-R	TGTAGACCATGTAGTTGAGGTCA
Jpx-m-F	TTAGCCAGGCAGCTAGAGGA
Jpx-m-R	GGAGAAATCCGGAGTGCCAG
Malat1-m-F	GGAAGTTGTTGGGGTGGGAT
Malat1-m-R	GTCAAGCAAAGACACCGCAG
Rabep1-m-F	CTTCGTTCTGTCGTGATGCC
Rabep1-m-R	ACTGGTTGTGTTGTTGTCGTT
Ireb2-m-F	TTCTGCCTTACTCAATACGGGT
Ireb2-m-R	AGGGCACTTCAACATTGCTCT
Tank-m-F	AGACATAGTCTGCGAAGGAACG
Tank-m-R	ATGCTCTATTGAGTTGCTCACC
Tbk1-m-F	ACTGGTGATCTCTATGCTGTCA
Tbk1-m-R	TTCTGGAAGTCCATACGCATTG
Chuk-m-F	GTCAGGACCGTGTTCTCAAGG
Chuk-m-R	GCTTCTTTGATGTTACTGAGGGC
Ecd-m-F	ACACCACCAACAATCCAGCA
Ecd-m-R	ATGCTTCGACCCTTTCTGGG
